# Epigallocatechin-3-Gallate (EGCG)-Inducible SMILE Inhibits STAT3-Mediated Hepcidin Gene Expression

**DOI:** 10.3390/antiox9060514

**Published:** 2020-06-11

**Authors:** Yu-Ji Kim, Ki-Sun Kim, Daejin Lim, Dong Ju Yang, Jae-Il Park, Ki Woo Kim, Jae-Ho Jeong, Hueng-Sik Choi, Don-Kyu Kim

**Affiliations:** 1 Department of Integrative Food, Bioscience and Biotechnology, Chonnam National University, Gwangju 61186, Korea; call7502@naver.com; 2 School of Biological Sciences and Technology, Chonnam National University, Gwangju 61186, Korea; kkscorea@naver.com (K.-S.K.); hsc@chonnam.ac.kr (H.-S.C.); 3 Department of Microbiology, Chonnam National University Medical School, Gwangju 61468, Korea; kumdoman7@hanmail.net (D.L.); jeongjaeho@chonnam.ac.kr (J.-H.J.); 4 Department of Oral Biology, BK21 PLUS, Yonsei University College of Dentistry, Seoul, 03722, Korea; YDJ1991@yuhs.ac (D.J.Y.); KIWOO-KIM@yuhs.ac (K.W.K.); 5 Korea Basic Science Institute, Gwangju Center at Chonnam National University, Gwangju 61186, Korea; jaeil74@kbsi.re.kr

**Keywords:** epigallocatechin-3-gallate, SMILE, IL-6, STAT3, FoxO1, hepcidin, iron metabolism, anemia of chronic disease

## Abstract

Hepatic peptide hormone hepcidin, a key regulator of iron metabolism, is induced by inflammatory cytokine interleukin-6 (IL-6) in the pathogenesis of anemia of inflammation or microbial infections. Small heterodimer partner-interacting leucine zipper protein (SMILE)/CREBZF is a transcriptional corepressor of nuclear receptors that control hepatic glucose and lipid metabolism. Here, we examined the role of SMILE in regulating iron metabolism by inflammatory signals. Overexpression of SMILE significantly decreased activation of the Janus kinase 2-signal transducer and activator of transcription 3 (STAT3)-mediated hepcidin production and secretion that is triggered by the IL-6 signal in human and mouse hepatocytes. Moreover, SMILE co-localized and physically interacted with STAT3 in the nucleus in the presence of IL-6, which significantly suppressed binding of STAT3 to the hepcidin gene promoter. Interestingly, epigallocatechin-3-gallate (EGCG), a major component of green tea, induced SMILE expression through forkhead box protein O1 (FoxO1), as demonstrated in FoxO1 knockout primary hepatocytes. In addition, EGCG inhibited IL-6-induced hepcidin expression, which was reversed by SMILE knockdown. Finally, EGCG significantly suppressed lipopolysaccharide-induced hepcidin secretion and hypoferremia through induction of SMILE expression in mice. These results reveal a previously unrecognized role of EGCG-inducible SMILE in the IL-6-dependent transcriptional regulation of iron metabolism.

## 1. Introduction

Anemia of inflammation (AI), also called anemia of chronic disease, is the most common type of anemia, following iron deficiency anemia [1]. The major etiologies of AI are acute and chronic infections, autoimmune disorders, chronic kidney disease, malignancies and inflammation. The pathogenesis of AI is given through reduction in the lifespan of erythrocytes, impaired proliferation of erythroid progenitor cells and iron accumulation in the cells of the mononuclear phagocytic system by production of pro-inflammatory cytokines such as tumor necrosis factor α and interleukin-6 (IL-6) causing hypoferremia [1,2]. Iron is absorbed into the enterocytes, circulates through blood in a form of transferrin-bound iron and transports into another organs and tissues for utilization. In addition, iron recycling from macrophages for erythropoiesis is also required to maintain iron homeostasis [3,4]. Hepcidin, a hepatic peptide hormone, plays a key role in iron metabolism by binding to the cell surface the iron transporter ferroportin (FPN) and triggering its internalization and degradation [5,6]. It is reported that hypoxia-inducible factors decrease hepcidin expression during hypoxia and erythropoiesis [7,8], while hepcidin is mainly induced by activation of the IL-6/Janus kinase 2 (JAK2)/signal transducer and activator of transcription 3 (STAT3) signaling pathway in the pathogenesis of AI or microbial infections [4,9].

Epigallocatechin-3-gallate (EGCG), a type of polyphenol, is the richest and most potent catechin in green tea [10]. EGCG has many beneficial biological functions, such as anti-inflammatory, anti-carcinogenic and anti-oxidative, and neuroprotective functions and cholesterol-lowering effects, according to concentration, cell type and other factors. EGCG regulates numerous intracellular signaling pathways by interacting with membrane receptors, activating second messengers and modulating various transcription factors and metabolic enzymes and finally stimulating cellular protective systems [10,11]. In addition, it was reported that EGCG induces epithelial specific erythroblast transformation-specific transcription factor-1 expression, resulting in suppression of colorectal tumorigenesis [12]. In hepatoma cells, EGCG improves insulin sensitivity via reducing the levels of circulating free fatty acids, inflammation and lipotoxicity, mediated by glucose and palmitic acid [13]. However, whether there is a biological function of EGCG in iron metabolism is largely unknown.

Small heterodimer partner (SHP)-interacting leucine zipper protein (SMILE)/CREBZF belongs to the cAMP response element-binding and activating transcription factor (CREB/ATF) family of the basic region-leucine zipper (bZIP) family and could homodimerize, but it lacks the binding activity to DNA as a homodimer [14]. It is reported that curcumin, a major polyphenol found in turmeric, induces SMILE expression in hepatocytes and modulates expression of the endoplasmic reticulum stress-regulated target gene by suppressing the transcriptional activity of cAMP responsive element-binding protein H [15]. Interestingly, insulin-inducible SMILE suppresses hepatic gluconeogenesis by down-regulating the expression of gluconeogenic genes such as phosphoenolpyruvate carboxykinase and glucose-6-phosphatase and ameliorates hyperglycemia in diabetes [16]. In addition, ursodeoxychoic acid-inducible SMILE relieves hepatic lipid accumulation through inhibiting expression of liver X receptor α-mediated lipogenic genes [17]. Therefore, SMILE has been considered as a corepressor with a critical role in liver metabolism [18,19,20]. However, the role of SMILE in iron metabolism has not yet been elucidated. To this end, we examined the effect of SMILE on IL-6-dependent regulation of hepcidin expression in hepatocytes and further investigated the polyphenol EGCG-inducible SMILE effect on altering the iron metabolism that is triggered by an IL-6 signal.

## 2. Materials and Methods

### 2.1. Chemicals

Recombinant human IL-6 (R&D Systems, Inc., Minneapolis, MN, USA) and EGCG (Tocris Bioscience, Bristol, UK) were dissolved in deionized water for in vitro studies and in phosphate buffered saline (PBS) for in vivo studies. Lipopolysaccharide (O111:B4 LPS, L2630) was purchased from Sigma-Aldrich (St. Louis, MO, USA). We used 20 ng/mL of IL-6 or 100 μM of EGCG for in vitro studies and 100 mg/kg of EGCG or 1 mg/kg of LPS for in vivo studies, unless otherwise stated.

### 2.2. Plasmid DNAs and Recombinant Adenoviruses

Mouse (mHepcidin-luc, −982/+84 bp) and human (hHepcidin-luc, −2762 bp) hepcidin gene promoters were indicated previously [21,22]. TEL-JAK2, FLAG-STAT3 and constitutively active STAT3 (STAT3-c) were described previously [22,23]. Mouse (mSMILE-luc, −1750/+126 bp) and human SMILE gene promoter (hSMILE-luc, −1006/+117 bp) were PCR-amplified from mouse and human genomic DNA, respectively, and then inserted into the pGL3 basic vector using *Sma* I and *Mlu* I restriction enzyme sites for the mouse SMILE gene promoter and *Xho* I and *Mlu* I restriction enzyme sites for the human SMILE gene promoter. pcDNA3-FLAG-hSMILE, pcDNA3-HA-hSMILE and pEGFP-hSMILE were indicated previously [18]. pCMV-FLAG-FoxO1 was kindly provided from Dr. Tadahiro Kitamura (Gunma University). Adenovirus-expressing green fluorescent protein (GFP) (Ad-GFP) and SMILE (Ad-SMILE) were described previously [18]. Ad-FoxO1 was purchased from Applied Biological Materials (abm; Richmond, BC, Canada).

### 2.3. Animal Experiments

Female 8-week-old C57BL/6J mice (Jackson Laboratory, Bar Harbor, ME, USA) were used for this study. For liver-specific FoxO1 knockout (FoxO1-LKO) studies, C57BL/6J mice containing floxed FoxO1 (FoxO1^f/f^) were obtained from Dr. Ronald A. DePinho (University of Texas MD Anderson Cancer Center). To produce the liver-specific FoxO1 knockout line (FoxO1-LKO), FoxO1^f/f^ animals were crossbred with C57BL/6J-Alb-Cre transgenic mice, which express Cre recombinase in hepatocytes under the control of the albumin promoter (Jackson Laboratory). All mice were acclimatized to a 12 h light/dark cycle at 22 ± 2 °C with free access to food and water in a specific pathogen-free facility. To investigate the EGCG effect on LPS-dependent hepcidin expression, we carried out intraperitoneal injection of PBS (*n* = 5), EGCG (*n* = 5), LPS (*n* = 7) and EGCG plus LPS (*n* = 6). Mice were injected with LPS for 12 h after 2 h of the PBS and EGCG injection. All experimental procedures were reviewed and approved by the Institutional Animal Care and Use Committee of Chonnam National University (CNU IACUC-H-2019-14).

### 2.4. Cell Culture, Transfection and Luciferase Assay

HepG2 (human liver cancer cell line; ATCC, Manassas, VA, USA) cells were cultured in Dulbecco’s modified Eagle’s medium (DMEM, high glucose, Welgene, Gyeongsangbuk-do, Korea), supplemented with 10% fetal bovine serum (FBS; Gibco, USA) and 1% antibiotics (penicillin-streptomycin; CAPRICORN SCIENTIFIC, Ebsdorfergrund, Germany). Huh7 (human hepatoma cell line, ATCC) cells were cultured in RPMI 1640 medium (Welgene) supplemented with 10% FBS and 1% antibiotics. AML12 (mouse hepatocyte cell line, ATCC) cells were cultured in DMEM/F-12 medium (Welgene) supplemented with 10% FBS, 1% insulin-transferrin-selenium-pyruvate supplement (Welgene), 40 ng/mL dexamethasone and 1% antibiotics. All the cell lines were cultured in humidified air containing 5% CO2 at 37 °C. Transient transfections were carried out using polyethylenimine (Polysciences, Inc., Warrington, PA, USA) or SuperFect (QIAGEN, Hilden, Germany), according to manufacturer’s instruction. Luciferase assay was performed as described previously [24]. Briefly, cells were transfected with indicated reporter plasmids along with expression vectors encoding SMILE, JAK2, STAT3-c and FoxO1 or treated with IL-6 or EGCG for 12 h. Total DNA used for each transfection was adjusted to 2 μg/well by adding an appropriate amount of empty vector, and Nano-Glo plasmid (Promega, Madison, WI, USA) was used as an internal control. The firefly luciferase activity was normalized to the Nano-Glo luciferase activity. Adenoviral infections (multiplicity of infection, MOI) or IL-6 and EGCG treatment were performed as described in figure legends.

### 2.5. Culture of Mouse Primary Hepatocytes

Mouse primary hepatocytes were isolated from wild-type (WT) and FoxO1-LKO mice (male or female, 20–25 g) by collagenase perfusion using a previously indicated method [25]. Primary hepatocytes were cultured in high-glucose DMEM supplemented with 10% FBS and 1% antibiotics. After attachment, the cells were treated with IL-6 and EGCG for 12 h.

### 2.6. Quantitative PCR

Total RNA was extracted from cultured hepatocytes or mice liver tissue using Tri-RNA Reagent (Favorgen Biotech Corporation, Ping-Tung, Taiwan), according to the manufacturer’s instructions. Quantity and purity of the RNAs were confirmed using a Biophotometer D30 (Eppendorf, Hamburg, Germany). RNAs were reverse-transcribed into cDNAs using the TOPscript RT DryMIX dT18plus (Enzynomics, Daejeon, Korea) following the manufacturer’s recommendations. Quantitative PCR (Q-PCR) was performed on a CFX Connect real-time system (Bio-Rad, Hercules, CA, USA) using the TOPreal qPCR 2X PreMIX (Enzynomics). The mRNA levels were normalized to L32 (forward: 5′-TCTGGTGAAGCCCAAGATCG- 3′, reverse: 5′-CTCTGGGTTTCCGCCAGT-3′) or β-actin (forward: 5′-GACAGGATGCAGAAGGAGATTAC- 3′, reverse: 5′-GCTGATCCACATCTGCTGGAA-3′) gene expression and variations and relative gene expressions were analyzed using a cycle threshold (delta-delta Ct) method.

### 2.7. Western Blot Analysis

Whole-cell extracts of mouse livers and cell lines or mouse primary hepatocytes were prepared using RIPA buffer (Thermo Fisher Scientific, Waltham, MA, USA) as previously described [26]. Proteins from whole-cell lysates were separated by 10–12% SDS-PAGE and transferred to nitrocellulose or PVDF membranes (EMD Millipore, MA, USA). The membranes were probed with following primary antibodies: anti-OctA (FLAG; Santa Cruz Biotechnology, Dallas, TX, USA), anti-HA (Santa Cruz Biotechnology), anti-β-actin (Santa Cruz Biotechnology), anti-Ferroportin (Novus Biologicals, Centennial, CO, USA), anti-SMILE (Zhangfei; Abcam, Cambridge, UK), anti-FoxO1 and anti-phospho-FoxO1 (Ser256) (Cell signaling Technology, MA, USA). Primary antibodies were detected using HRP-conjugated secondary antibodies. All antibodies were used at a dilution of 1:1000 to 1:3000. Images were acquired using Amersham ECL reagents (GE Healthcare, IL, USA) and a ChemiDoc XRS system (Bio-Rad).

### 2.8. Co-immunoprecipitation Analysis

Co-immunoprecipitation analysis was conducted using a FLAG immunoprecipitation kit (Sigma) according to the manufacturer’s protocol. Briefly, HepG2 cells were transfected with vectors expressing HA-SMILE and FLAG-STAT3 and treated with IL-6 for 12 h. 200 μL of whole-cell lysates were prepared with IP lysis buffer (Thermo Fisher Scientific) and immunoprecipitated with anti-FLAG M2 affinity gel for 16 h. After the immunoprecipitation, proteins eluted with 3X FLAG peptide were analyzed with WB using anti-HA and anti-FLAG antibody (Santa Cruz Biotechnology).

### 2.9. Immunocytochemistry

HepG2 cells were transfected with vectors expressing GFP-SMILE and FLAG-STAT3 and treated with IL-6 for 12 h. The cells were processed for immunocytochemical assay using Alexa Fluor SFX Kits (Molecular Probes, Invitrogen, CA, USA). Immunofluorescence staining for FLAG-STAT3 was carried out using DYKDDDDK Tag polyclonal antibody (1:200 dilution, Invitrogen) and Alexa Fluor 594-conjugated goat anti rabbit secondary antibody (1:200 dilution, Invitrogen). The cells were mounted with DAPI (4′,6-diamidino-2-phenylindole)-containing reagent (ProLong Gold antifade reagent with DAPI, Molecular Probes, Invitrogen) and observed with a laser-scanning confocal microscope (Leica TCS SP5/Tandem/AOBS, Leica Microscope Systems, Wetzlar, Germany) in Korea Basic Science Institute (Gwangju Center, Korea).

### 2.10. Chromatin Immunoprecipitation Assay

The chromatin immunoprecipitation (ChIP) assay was conducted according to the manufacturer’s protocol (Cell Signaling Technology). Briefly, HepG2 cells were transfected with indicated plasmid constructs and treated with IL-6 for 12 h. Cells were fixed with 1% formaldehyde and then harvested. The soluble chromatin was immunoprecipitated with anti-FLAG M2 affinity gel (Sigma). After DNA was recovered, Q-PCR was performed using the STAT3-RE (forward: 5′-GAGCCACAGTGTGACATCAC-3′, reverse: 5′-GTCTAGGAGCCAGTCCCAGT-3′) and FoxO1-binding site (rorward: 5′-GCCTAAGTAACTACCATGACTT-3′, reverse: 5′-GTCATTATTACATCATTTTTA-3′) primers.

### 2.11. Serum Iron and Hepcidin Measurement

All blood samples were taken using an intra-cardiac puncture of mice under anesthesia before killing. Serum iron was measured using a spectrophotometric method (TBA-200FR NEO). Serum hepcidin was measured using a Mouse Hepc (Hepcidin) ELISA kit (Elabscience, TX, USA), according to the manufacturer’s protocols. For hepcidin measurement in cell culture media, Huh7 or AML12 cells were transfected with vector expressing SMILE and treated with IL-6 for 12 h after 2 h of serum starvation. Hepcidin levels were measured from the cell culture media using a Mouse Hepc (Hepcidin) ELISA kit (Elabscience) and a Human Hepcidin Quantikine ELISA kit (R&D Systems) according to the manufacturer’s protocols.

### 2.12. RNA Interference

Small interfering RNAs against control (si-Con) and human SMILE (si-SMILE) were purchased from QIAGEN (Cat. No. 1027416). HepG2 cells were transfected with si-Con and si-SMILE using Lipofectamine RNAi MAX (Thermo Fisher Scientific) according to the manufacturer’s instruction.

### 2.13. Cell Viability

HepG2 cells were treated with EGCG for 0 h, 6 h, 12 h and 24 h and incubated with MTT (final concentration: 0.5 mg/mL) for 2 h at 37 °C. The medium was removed, and the insoluble formazan product was dissolved in dimethyl sulfoxide. The absorbance was measured at 560 nm using a microplate reader (Biotek, Winooski, VT, USA). The absorbance of control cells was considered to represent 100% viability.

### 2.14. Statistical Analysis

Statistical analyses were performed using GraphPad Prism (GraphPad Software, CA, USA). The data are presented as the means ± SD or ± SEM. Statistical significance was estimated using a two-tailed Student’s *t*-test. Differences were considered statistically significant at *P* < 0.05.

## 3. Results

### 3.1. SMILE Inhibits IL-6-Mediated Hepcidin Production and Secretion

**Figure 1:**
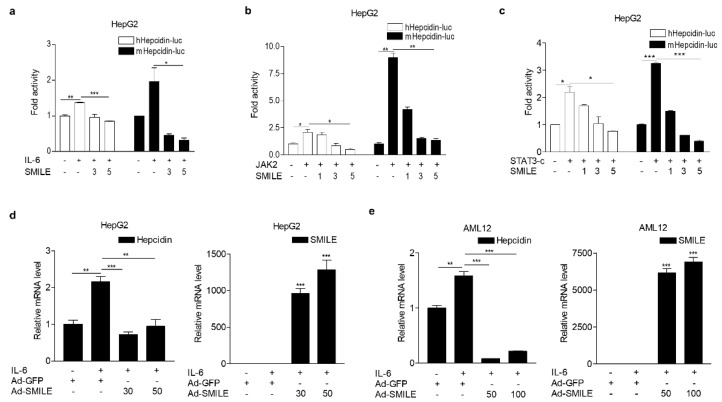
SMILE inhibits hepcidin expression by IL-6 signal. (**a**) Effect of SMILE on IL-6-induced hepcidin promoter activity. HepG2 cells were transfected with expression vectors encoding hepcidin promoters (hHpeicidn-luc; 100 ng or mHepcidin-luc; 200 ng) and pcDNA3-FLAG-hSMILE (3, 300 ng; 5, 500 ng) and then treated with IL-6 (20 ng/mL) for 12 h. (**b**) Effect of SMILE on JAK2-mediated hepcidin promoter activity. HepG2 cells were transfected with expression vectors encoding hepcidin promoters, JAK2 (+, 200 ng with hHepcidin-luc or 400 ng with mHepcidin-luc) and pcDNA3-FLAG-hSMILE (1, 100 ng; 3, 300 ng; 5, 500 ng). (**c**) Effect of SMILE on STAT3-induced hepcidin promoter activity. HepG2 cells were transfected with expression vectors encoding hepcidin promoters, STAT3-c (+, 200 ng with hHepcidin-luc or 500 ng with mHepcidin-luc) and pcDNA3-FLAG-hSMILE (1, 100 ng; 3, 300 ng; 5, 500 ng). (**d–e**) Inhibitory effect of SMILE on hepcidin mRNA expression. HepG2 cells (**d**) and AML12 cells (**e**) were infected with Ad-GFP (50, 100 MOI) or Ad-SMILE (30, 50, 100 MOI) for 36 h and then treated with IL-6 (20 ng/mL) for 12 h. All experiments were performed in duplicate or triplicate and repeated at least three times. The values are presented as means ± SD. * *P* < 0.05, ** *P* < 0.01, *** *P* < 0.001 using two-tailed Student’s *t*-test.

**Figure 2:**
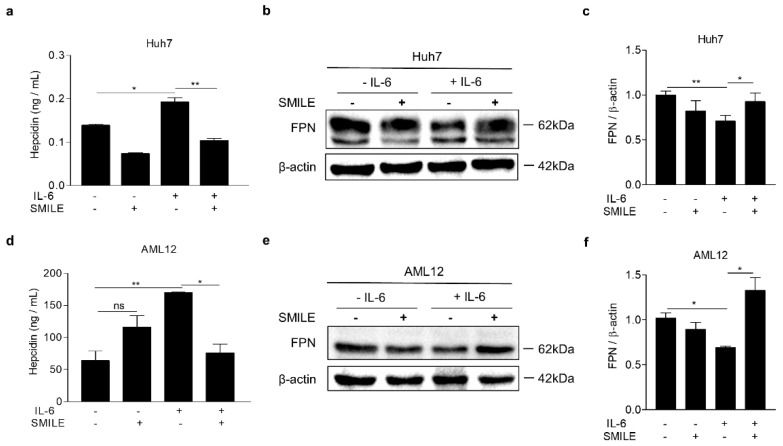
SMILE suppresses IL-6-induced hepcidin secretion. (**a**–**f**) Huh7 and AML12 cells were transfected with vector encoding SMILE (5 μg) and treated with IL-6 for 12 h after 2 h of serum starvation. Secreted hepcidin was measured using ELISA from culture medium. Ferroportin (FPN) expression was analyzed using western blot. Hepcidin secretion (**a**), FPN expression (**b**) and graphical representation (**c**) in Huh7 cells. Hepcidin secretion (**d**), FPN expression (**e**) and graphical representation (**f**) in AML12 cells. All gels for western blot analysis in (**b**,**e**) were run under the same experimental conditions including equal amounts (100 μg) of protein. The independent experiments were repeated at least three times. The values are presented as means ± SD. ns; not significant. * *P* < 0.05, ** *P* < 0.01 using two-tailed Student’s *t*-test.

It is reported that IL-6 increases hepcidin expression through activation of the JAK2-STAT3 signaling pathway in response to inflammation [27]. To examine the effect of SMILE on IL-6-induced hepcidin expression in hepatocytes, we performed transient transfections with vectors expressing SMILE and hepcidin gene promoter in HepG2 cells, a human hepatoma cell line, and treated with IL-6. Interestingly, IL-6-induced promoter activity of human and mouse hepcidin gene was significantly decreased by SMILE (Figure 1a). In addition, SMILE inhibited hepcidin gene promoter activity by JAK2 and STAT3, respectively (Figure 1b,c). To further examine the inhibitory action of SMILE on IL-6-induced hepcidin gene transcription, we conducted adenoviral overexpression of GFP (Ad-GFP) or SMILE (Ad-SMILE) in HepG2 and AML12 cells, a non-transformed mouse liver cell line, treated with IL-6. Consistent with these results in transient transfection, overexpression of SMILE significantly suppressed induction of hepcidin mRNA expression by IL-6 stimulation in both cell lines (Figure 1d,e). Furthermore, we also found that SMILE inhibited IL-6-mediated hepcidin secretion in both human and mouse hepatoma cells (Figure 2a,d). Consequently, SMILE normalized decreased ferroportin expression by hepcidin (Figure 2b,c,e,f). These findings indicated that SMILE functions as a repressor of hepcidin transcriptional control by IL-6 signaling.

### 3.2. SMILE Represses Activity of The IL-6 Signaling Pathway via Inhibition of DNA-Binding of STAT3

**Figure 3:**
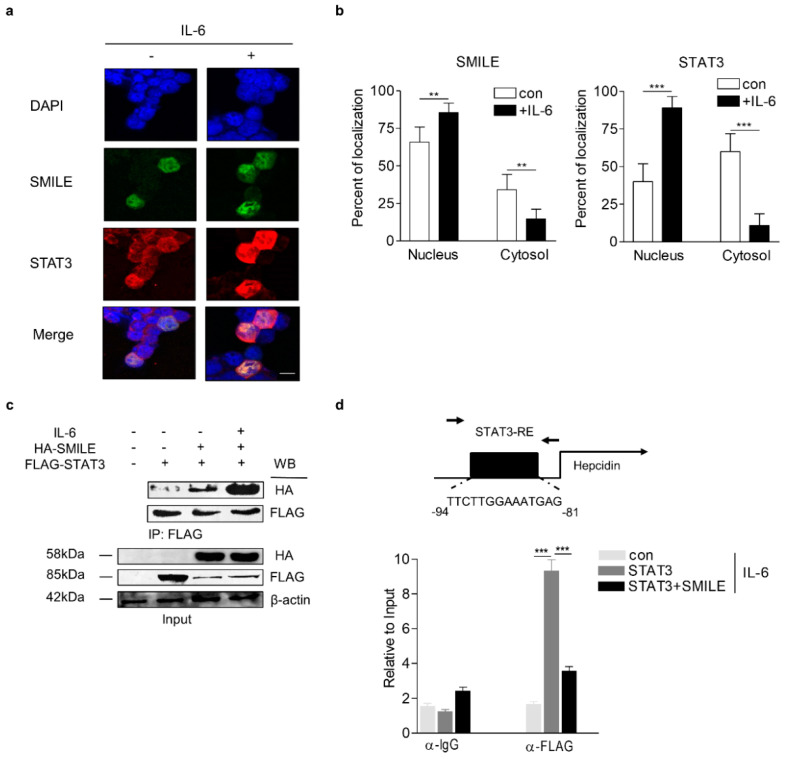
SMILE interacts with and represses STAT3 binding to hepcidin promoter. (**a**) Subcellular co-localization of SMILE and STAT3. HepG2 cells were co-transfected with vectors encoding GFP- SMILE and FLAG STAT3 and treated with IL-6 for 12 h. Scale bar shows 10 μm. (**b**) Relative fluorescence of SMILE (left) and FoxO1 (right) between nucleus and cytoplasm. (**c**) Co-immunoprecipitation analysis showing interaction between SMILE and STAT3. HepG2 cells were transfected with vectors encoding HA-SMILE and FLAG-STAT3 and treated with IL-6 for 12 h. Gels for western blot analysis were run under the same experimental conditions. (**d**) ChIP assay showing inhibitory effects of SMILE on STAT3 binding activity to hepcidin promoter. HepG2 cells were transfected with vectors encoding hepcidin promoter, HA-SMILE and FLAG-STAT3 and then treated with IL-6 for 12 h. Soluble chromatin was immunoprecipitated using anti-FLAG antibody. ChIP signals were measured using Q-PCR. STAT3-RE, STAT3-response element. The values are presented as means ± SD. ** *P* < 0.01, *** *P* < 0.001 using two-tailed Student’s *t*-test.

To identify the molecular mechanism by which SMILE suppresses IL-6-mediated hepcidin expression, we first investigated subcellular localization of SMILE and STAT3 in HepG2 cells treated with IL-6. While STAT3 was mainly localized in cytoplasm in the absence of IL-6, SMILE was localized in the nucleus. However, they co-localized in the nucleus in the presence of IL-6 (Figure 3a,b). In addition, co-immunoprecipitation analysis demonstrated that IL-6 strongly increased the interaction between SMILE and STAT3 (Figure 3c). Moreover, chromatin immunoprecipitation (ChIP) assay showed that STAT3 binds to a STAT3-responsive element (STAT3-RE) on the hepcidin promoter in the presence of IL-6, which was almost entirely abrogated by overexpression of SMILE (Figure 3d). These results suggest that SMILE disrupts IL-6-mediated hepcidin expression by inhibiting STAT3 binding to the hepcidin promoter.

### 3.3. EGCG Increases FoxO1 and SMILE Expression

**Figure 4:**
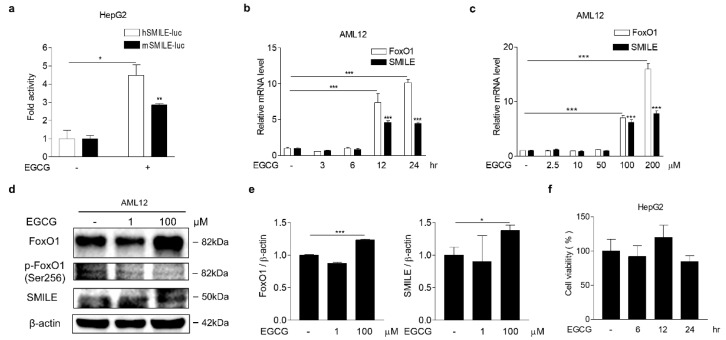
EGCG induces SMILE and FoxO1 expression. (**a**) EGCG effect on human and mouse SMILE promoter activity. HepG2 cells were transfected with vectors encoding human or mouse SMILE-luc (300 ng) and then treated with EGCG (100 μM) for 12 h. (**b**,**c**) Time- and dose-dependent mRNA expression of FoxO1 and SMILE by EGCG. AML12 cells were treated with EGCG (100 μM) for the indicated time (**b**) and dose (**c**) after 2 h of serum starvation. (**d**) Western blot analysis showing FoxO1, p-FoxO1 and SMILE expression in AML12 cells treated with EGCG for 12 h. Gels for western blot analysis were run under the same experimental conditions. (**e**) Graphical representation of FoxO1 (left) and SMILE (right). (f) Cell viability measured using MTT assay. HepG2 cells were treated with EGCG (100 μM) in a time-dependent manner. The independent experiments were repeated at least twice. The values are presented as means ± SD. * *P* < 0.05, *** *P* < 0.001 using two-tailed Student’s *t*-test.

The transcriptional repressive activity of SMILE is modulated by natural polyphenolic compounds such as curcumin, a major active component of turmeric, and resveratrol, a polyphenol found in red wine [15,20]. However, the effect of EGCG, a major polyphenol in green tea, on SMILE expression has not yet been elucidated. To this end, we examined SMILE gene promoter activity and mRNA expression in hepatocytes treated with EGCG for 12 h. As anticipated, we found that both human and mouse SMILE gene promoter activity were significantly induced by EGCG treatment (Figure 4a). In addition, mRNA expression of SMILE and forkhead box protein O1 (FoxO1), a downstream transcription factor of EGCG [28], was continuously increased with time in EGCG-treated AML12 cells (Figure 4b). Moreover, FoxO1 and SMILE mRNA levels were induced at high doses of EGCG (>100 μM) (Figure 4c). Indeed, FoxO1 and SMLE protein levels were induced in AML12 cells treated with 100 μM of EGCG for 12 h, while p-FoxO1 levels were unaltered at this condition (Figure 4d,e). Finally, to examine a cytotoxic effect of EGCG, we treated HepG2 cells with 100 μM of EGCG in a time-dependent manner and found that EGCG had no cytotoxic effect on HepG2 cells with 24 h of treatment (Figure 4f).

### 3.4. FoxO1 Increases SMILE Expression

**Figure 5:**
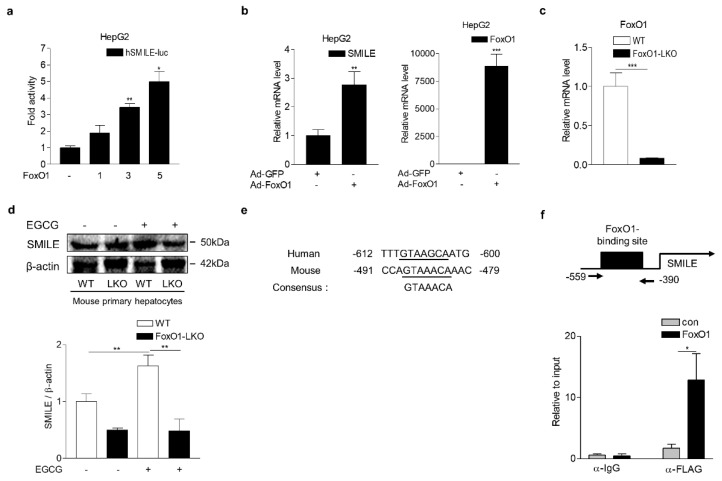
FoxO1 upregulates SMILE expression in hepatocytes (**a**) SMILE promoter activity by FoxO1. HepG2 cells were transfected with vectors encoding hSMILE-luc and FoxO1 (1, 100 ng; 3, 300 ng; 5, 500 ng). (**b**) FoxO1 increases SMILE mRNA expression. HepG2 cells were infected with Ad-GFP (10 MOI) or Ad-FoxO1 (10 MOI) for 48 h. (**c**) Q-PCR analysis showing the efficiency of FoxO1 knockdown in primary hepatocytes isolated from WT and FoxO1-LKO mice. (**d**) EGCG effect on SMILE protein levels in primary hepatocytes isolated from WT and FoxO1-LKO mice. Mouse primary hepatocytes were treated with EGCG (100 μM) for 12 h. Gels for western blot analysis were run under the same experimental conditions. (**e**) Alignment of FoxO1 binding sequences on human and mouse SMILE promoter. Underlined letters indicate consensus FoxO1 binding sequences. (**f**) ChIP assay showing FoxO1 binding to SMILE promoter. HepG2 cells were transfected with vectors encoding SMILE promoter and FLAG-FoxO1. Soluble chromatin was immunoprecipitated using anti-FLAG antibody. ChIP signals were measured using Q-PCR. The independent experiments were repeated at least twice. The values are presented as means ± SD. * *P* < 0.05, ** *P* < 0.01, *** *P* < 0.001 using two-tailed Student’s *t*-test.

Next, we tested if FoxO1 regulates SMILE gene transcription using wild-type (WT) and liver-specific FoxO1 knockout (FoxO1-LKO) hepatocytes. Overexpression of FoxO1 significantly induced SMILE gene promoter activity and mRNA expression (Figure 5a,b). In addition, EGCG treatment increased SMILE protein levels in WT hepatocytes but not in FoxO1-LKO hepatocytes (Figure 5c,d). Moreover, close investigation of the SMILE gene promoter revealed that a putative FoxO1 binding sequence was conserved in human and mouse (Figure 5e). Consistent with this investigation, FoxO1 was significantly recruited to the FoxO1-binding region of the SMILE gene promoter, as investigated using ChIP assay (Figure 5f). These findings indicate that FoxO1 is a direct transcriptional regulator of SMILE gene in the presence of EGCG.

### 3.5. EGCG Attenuates IL-6-Induced Hepcidin Expression through SMILE

**Figure 6:**
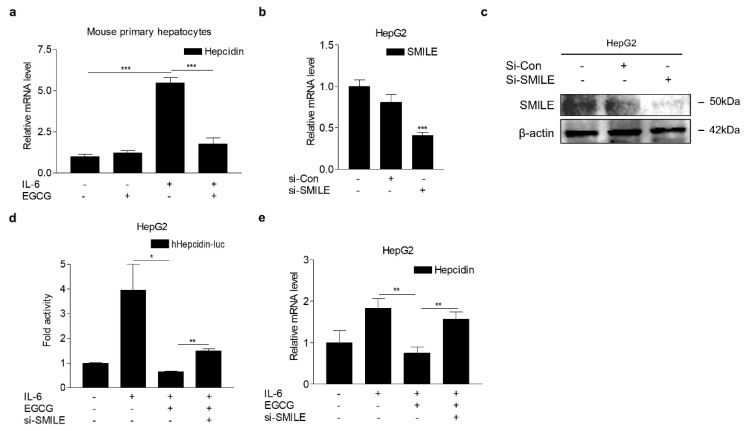
Effects of SMILE knockdown on inhibiting hepcidin expression by EGCG. (**a**) EGCG effect on IL-6-induced hepcidin expression in mouse primary hepatocytes treated with IL-6 (20 ng/mL) and EGCG (100 μM) for 12 h. (**b**) Q-PCR analysis showing efficiency of SMILE knockdown. HepG2 cells were transfected with si-Con and si-SMILE for 48 h. (**c**) Western blot analysis showing efficiency of SMILE knockdown. HepG2 cells were transfected with si-Con and si-SMILE for 66 h. (**d**) Transient transfection analysis showing the effect of SMILE knockdown on EGCG-reduced hepcidin promoter activity. HepG2 cells were transfected with Hepcidin-luc (200 ng) and si-SMILE and then treated with IL-6 (20 ng/mL) and EGCG (100 μM) for 12 h. (**e**) Q-PCR analysis showing SMILE knockdown effect on EGCG-mediated inhibition of hepcidin expression. HepG2 cells were transfected with si-SMILE and then treated with IL-6 (20 ng/mL) and EGCG (100 μM) for 12 h. The independent experiments were repeated at least twice. The values are presented as means ± SD. * *P* < 0.05, ** *P* < 0.01, *** *P* < 0.001 using two-tailed Student’s *t*-test.

In order to investigate the role of SMILE in EGCG action on regulation of hepcidin expression, we first examined the effect of EGCG on IL-6-mediated hepcidin expression in mouse primary hepatocytes. As expected, IL-6 significantly increased hepcidin mRNA expression, which was almost entirely reduced by EGCG (Figure 6a). Next, to demonstrate the role of SMILE in inhibiting hepcidin expression by EGCG, we treated HepG2 cells with IL-6 and EGCG after transfection of vector-carrying hepcidin gene promoter and SMILE knockdown using small interfering RNAs for SMILE (si-SMILE). SMILE mRNA and protein levels were significantly decreased by si-SMILE (Figure 6b,c). Consequently, the inhibitory effect of EGCG on IL-6-induced hepcidin promoter activity was significantly blocked by SMILE knockdown (Figure 6d). In addition, we observed that SMILE knockdown considerably reversed the effect of the EGCG treatment on IL-6-mediated induction of hepcidin mRNA expression (Figure 6e). These results demonstrated that SMILE is required for EGCG action on the IL-6-dependent transcriptional regulation of hepcidin gene expression.

### 3.6. EGCG Inhibits LPS-Induced Hepcidin Expression and Hypoferremia in Mice

**Figure 7:**
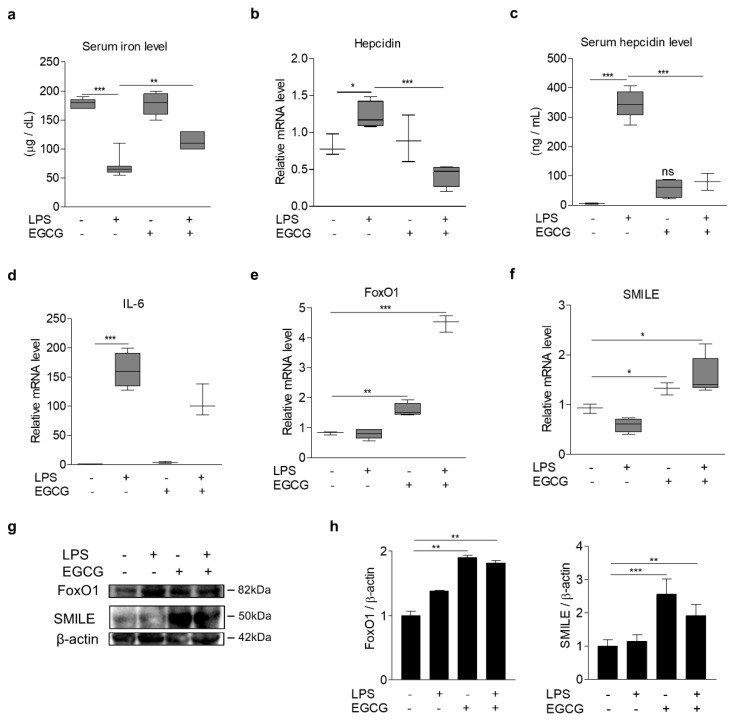
EGCG effects on LPS-induced iron metabolism in mice. (**a**–**h**) C57BL/6J mice were intraperitoneally injected with LPS (1 mg/kg, *n* = 7) for 12 h after 2 h of the PBS (*n* = 5) and EGCG (100 mg/kg, *n* = 5, EGCG-LPS: *n* = 6) injection. Serum iron levels (**a**). Hepcidin mRNA levels in liver (**b**). Serum hepcidin levels (**c**). IL-6, FoxO1 and SMILE mRNA levels in liver (**d**–**f**). Western blot analysis showing FoxO1 and SMILE expression (**g**). Graphical representation of FoxO1 (right) and SMILE (left) (**h**). mRNA levels were measured using Q-PCR. Gels for western blot analysis were run under the same experimental conditions. The values are presented as means ± SEM in (**a**–**f**) and ±SD in h. ns; not significant (Con vs. EGCG). * *P* < 0.05, ** *P* < 0.01, *** *P* < 0.001 using two-tailed Student’s *t*-test.

**Figure 8:**
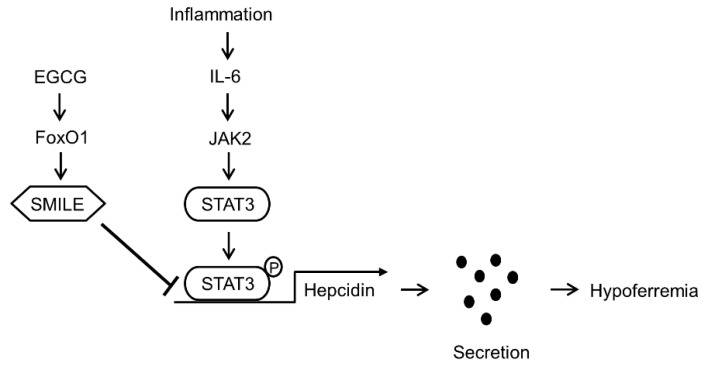
A model for SMILE function in IL-6-mediated hepcidin expression. SMILE expression is increased by EGCG-mediated induction of FoxO1 expression. In addition, SMILE expression inhibits STAT3 binding to hepcidin promoter and subsequently contributes to suppression of hepcidin production and secretion triggered by the IL-6 signal in hepatocytes.

Based on these findings in cultured cells, we next tested if EGCG suppresses hepatic hepcidin production and secretion by lipopolysaccharide (LPS), a key factor of IL-6 production [29]. As expected, intraperitoneal injection of LPS into mice caused a significant induction of IL-6 and hepcidin levels in liver and serum and a reduction of serum iron levels (Figure 7a–d). However, EGCG treatment significantly increased hepatic FoxO1 and SMILE expression (Figure 7e–h) and reversed the LPS effect on hepcidin and serum iron levels without significant changes in the IL-6 expression (Figure 7a–c). Taken together, these results suggest that EGCG-inducible SMILE acts as an important negative regulator for the IL-6-depedent induction of hepcidin expression (Figure 8).

## 4. Discussion

Iron homeostasis is maintained by the tight regulation of dietary iron absorption from enterocytes and release of iron from macrophages and hepatocytes. Hepcidin, an iron homeostatic hormone, is regulated through two major signaling pathways: inflammatory cytokine IL-6-dependent activation of JAK2-STAT3 signaling and excessive iron-mediated activation of bone morphogenetic protein-6 (BMP-6)-SMADs signaling [30]. Recently, we reported that SHP is a transcriptional corepressor of BMP-6-SMAD1/5/8-activated hepcidin expression triggered by an iron signal [31]. However, transcriptional corepressors of STAT3 in IL-6-mediated hepcidin induction have not yet been elucidated. In this study, we identified SMILE as a transcriptional corepressor of STAT3 suppressing hepcidin production and eventual hypoferremia. Indeed, the ChIP assay revealed that SMILE directly interacted with STAT3 and almost entirely blocked IL-6-induced STAT3 binding to hepcidin promoter. Previously, we also demonstrated that in response to *Salmonella typhimurium*, nuclear receptor estrogen-related receptor γ (ERRγ), known as a SMILE-interacting protein [20], mediates IL-6-induced hepcidin expression in mouse livers [22]. These results suggest that SMILE plays a critical role as transcriptional corepressor of the IL-6 signal in hepatocytes by inhibiting transcriptional activity of both STAT3 and ERRγ on hepcidin expression. In addition, it is reported that SMILE controls liver regeneration by inhibiting STAT3 activation in carbon tetrachloride-treated mice [32], indicating that the mechanism by which SMILE suppresses STAT3 activation could be applied for other cell physiologies.

EGCG, a major polyphenolic component of green tea, is known to have antioxidant effects by inducing expression of antioxidant enzymes including superoxide dismutase (SOD) and glutathione peroxidase. Interestingly, however, EGCG acts as a pro-oxidant by producing reactive oxygen species (ROS), such as hydrogen peroxide and hydroxyl radicals, at high concentrations (>50 μM) [10]. For example, EGCG activated AMP-dependent kinase (AMPK) by inducing ROS-dependent CaMKKβ and liver kinase B1 (LKB1) activity in hepatocytes, adipocytes and endothelial cells as well as cancer cells [33,34]. In this study, we found that EGCG increases SMILE gene expression at high concentration in hepatocytes, suggesting that the pro-oxidant effect of EGCG contributes to inducing SMILE expression. These findings are further supported by a previous report showing that curcumin, a polyphenol, significantly induces SMILE expression by activating the LKB1-AMPK pathway [15]. In addition, we showed that EGCG increases FoxO1 expression, resulting in induction of SMILE in hepatocytes. These findings are further supported by the results showing that EGCG-induced SMILE expression was almost entirely blocked in FoxO1-LKO hepatocytes. Recently, it is also reported that EGCG significantly increased FoxO1 expression in liver [28]. These results suggest that FoxO1 plays a critical role in EGCG-mediated SMILE induction. However, the antioxidant function of EGCG is reported to inhibit FoxO1 transcriptional activity by activating Akt, which phosphorylates FoxO1 in adipocytes [35]. In addition, it is reported that EGCG downregulates FoxO1 without affecting insulin signaling in hepatocytes [36]. These results indicate that FoxO1 activity and cellular redox state are intrinsically linked to each other because ROS modulates FoxO1 activity at the transcriptional and posttranslational levels, which in turn regulates expression of antioxidant proteins, such as SOD, peroxiredoxins and selenoprotein P, which contribute to cellular antioxidant defense [37]. Therefore, these findings suggest that FoxO1 might act as a cellular redox sensor linking between anti- and pro-oxidant functions of EGCG. However, the detailed mechanisms by which EGCG regulates FoxO1 in hepatocytes needs to be further characterized.

AI is a form of anemia found in several disease states such as chronic infections, chronic immune activation and malignancies. These conditions produce massive induction of IL-6, which elevates hepcidin production and secretion from the liver, which in turn promotes degradation of FPN and eventual hypoferremia due to reduced access of iron to the circulation [6]. Therefore, pharmacological control of IL-6-dependent hepcidin expression can provide a therapeutic approach to ameliorating AI. In the current study, we elucidated that SMILE acts as a transcriptional corepressor of IL-6-dependent hepcidin expression by inhibiting STAT3 binding to hepcidin promoter, leading to reduced hepcidin secretion from hepatocytes (Figure 8). In addition, EGCG increased SMILE expression through FoxO1 induction, which reversed the IL-6 effect on hepcidin production and eventual hypoferremia in mice. Taken together, these findings suggest that EGCG that induces SMILE expression would have potential for treating AI.

## Abbreviations

AI	Anemia of inflammation
EGCG	Epigallocatechin-3-gallate
SMILE	Small heterodimer partner-interacting leucine zipper protein
IL-6	Interleukin-6
JAK2	Janus kinase 2
STAT3	Signal transducer and activator of transcription 3
STAT3-c	STAT3 constitutive active form
STAT3-RE	STAT3-responsive element
FoxO1	Forkhead box protein O1
FPN	Ferroportin
LPS	Lipopolysaccharide
WT	Wild type mice
FoxO1-LKO	Liver-specific FoxO1 knockout mice
MOI	Multiplicity of infection
SHP	Small heterodimer partner
ERRγ	Estrogen-related receptor γ
ELISA	Enzyme-linked immunosorbent assay
Q-PCR	Quantitative PCR
BMP-6	Bone morphogenetic protein-6
ROS	Reactive oxygen species
LKB1	Liver kinase B1
AMPK	AMP-dependent kinase
